# Omics Approaches for Understanding Grapevine Berry Development: Regulatory Networks Associated with Endogenous Processes and Environmental Responses

**DOI:** 10.3389/fpls.2017.01486

**Published:** 2017-09-07

**Authors:** Alejandra Serrano, Carmen Espinoza, Grace Armijo, Claudio Inostroza-Blancheteau, Evelyn Poblete, Carlos Meyer-Regueiro, Anibal Arce, Francisca Parada, Claudia Santibáñez, Patricio Arce-Johnson

**Affiliations:** ^1^Laboratorio de Biología Molecular y Biotecnología Vegetal, Departamento de Genética Molecular y Microbiología, Pontificia Universidad Católica de Chile Santiago, Chile; ^2^Núcleo de Investigación en Producción Alimentaría, Facultad de Recursos Naturales, Escuela de Agronomía, Universidad Católica de Temuco Temuco, Chile; ^3^Ecophysiology and Functional Genomic of Grapevine, Institut des Sciences de la Vigne et du Vin, Institut National de la Recherche Agronomique, Université de Bordeaux Bordeaux, France

**Keywords:** grapevine fruit development, seed development, biotic and abiotic stresses, transcriptomics, metabolomics

## Abstract

Grapevine fruit development is a dynamic process that can be divided into three stages: formation (I), lag (II), and ripening (III), in which physiological and biochemical changes occur, leading to cell differentiation and accumulation of different solutes. These stages can be positively or negatively affected by multiple environmental factors. During the last decade, efforts have been made to understand berry development from a global perspective. Special attention has been paid to transcriptional and metabolic networks associated with the control of grape berry development, and how external factors affect the ripening process. In this review, we focus on the integration of global approaches, including proteomics, metabolomics, and especially transcriptomics, to understand grape berry development. Several aspects will be considered, including seed development and the production of seedless fruits; veraison, at which anthocyanin accumulation begins in the berry skin of colored varieties; and hormonal regulation of berry development and signaling throughout ripening, focusing on the transcriptional regulation of hormone receptors, protein kinases, and genes related to secondary messenger sensing. Finally, berry responses to different environmental factors, including abiotic (temperature, water-related stress and UV-B radiation) and biotic (fungi and viruses) stresses, and how they can significantly modify both, development and composition of vine fruit, will be discussed. Until now, advances have been made due to the application of Omics tools at different molecular levels. However, the potential of these technologies should not be limited to the study of single-level questions; instead, data obtained by these platforms should be integrated to unravel the molecular aspects of grapevine development. Therefore, the current challenge is the generation of new tools that integrate large-scale data to assess new questions in this field, and to support agronomical practices.

## Introduction

**FIGURE 1:**
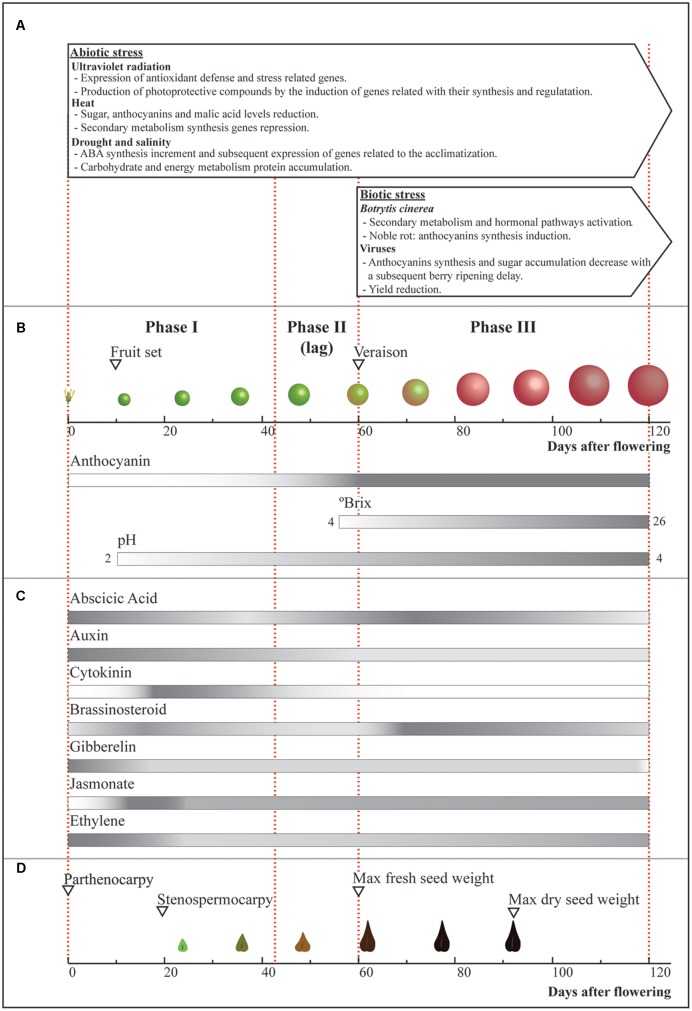
Fruit development and environmental effects. Scheme of the most important changes that berries and seeds undergo during development, and the main environmental factors affecting this process. **(A)** Boxes indicate the phase where each stress condition (temperature, water-related stress and UV-B radiation, *Botrytis cinerea* and viruses) affect grape berry and its development. **(B)** Changes in size, color, brix degree, and pH during berry ripening and **(C)** variations in hormonal content during grape berry development. **(D)** Seed development showing the time in which parthenocarpy and stenospermocarpy can take place. The main events are indicated by open triangles. Bars represent the described changes throughout development, in which gray and white represent the higher and lower estimated referential values for each parameter, respectively.

The grapevine (*Vitis vinifera*), one of the most important fruit crops worldwide, provides berries that can be used as fresh fruit, raisins, and for wine making and distillation of liquors. The grapevine has fleshy berries derived from the ovary of the flower, whose development is a complex process that can be divided into three stages with distinctive physiological and biochemical characteristics (Coombe and McCarthy, 2000). During the first stage (stage I) there is an exponential increase in berry size due to rapid cell division and growth, leading to the establishment of the final number of cells (Coombe and Hale, 1973). Some of the principal compounds that are present in the berry at stage I are tartaric and malic acids, which accumulate mainly in skin and flesh and confer acidity to fruits and wine (Sweetman et al., 2009, 2012). The second stage (stage II) is a lag phase in which important physiological and biochemical changes occur, such as softening and coloring. Within this stage, veraison takes place, characterized by the beginning of the synthesis of anthocyanins, soluble flavonoids compounds that provide color to red varieties (**Figure 1**) (Boss et al., 1996). Sucrose, originating from leaves, reaches the fruits through the phloem, and is then hydrolyzed forming glucose and fructose (Robinson and Davies, 2000; Kennedy, 2002; Vignault et al., 2005; Deluc et al., 2007; Hayes et al., 2007; Fontes et al., 2011). Stage II is thus a transition between an unripe fruit and the third stage of development (stage III or ripening). The latter involves important morphological and physiological changes, like color development (Boss et al., 1996), turgor reduction and berry enlargement (Chervin et al., 2008), and decreased acidity (Costenaro-da-Silva et al., 2010), among others. In addition, hormonal changes that occur throughout development positively or negatively regulate ripening (**Figure 1**; Gerós et al., 2012). Therefore, during ripening, a large number of complex transcriptional and/or post-transcriptional regulatory processes are triggered. In this review, we focus on the integration of global approaches, including proteomics, metabolomics, and especially transcriptomics, to understand grape berry development and the influence of environmental factors on this process. Thus, we will cover initial fruit development, with emphasis on seed formation; the importance of coloration and hormonal changes during development, especially on ripening; and finally, the effect of environmental factors on this process will be discussed.

## Grape Berry Development from an Omics Perspective

### Seed Development and Seedless Fruits

Grape berry development begins after fertilization, when in a process known as fruit set, the ovary changes from a stationary state and experiences an abrupt increase in size that occurs due to cell division and enlargement, leading to rapid pericarp growth. Throughout this period, seed development is an important process, mainly because seeds produce auxins, gibberellins (GAs) and cytokinins, which play multiple roles in grape berry development (Keller, 2010). Seed and berry development are coordinated, and the changes that seeds undergo have an impact on fruit ontogeny. The first stage of berry development is characterized by a rapid increase in seed size, during which embryogenesis and endosperm growth occur. At the second stage, about 10 and 15 days prior to veraison, seeds reach their final size and maximum fresh weight, and at the beginning of the third stage, embryo growth ceases and the endosperm accumulates reserves until the seeds turn dormant (**Figure 1**; Keller, 2010).

Ripe berries usually contain up to four seeds derived from four ovules (Dokoozlian, 2000; Kennedy, 2002). However, seedless grape varieties have been spontaneously found in nature and have been preserved over the years through asexual propagation. Seedless berries develop naturally via two different mechanisms, parthenocarpy and stenospermocarpy, which generate berries without or with rudimentary seeds, respectively (Varoquaux et al., 2000). In order to understand the main differences between parthenocarpy and stenospermocarpy, we will discuss the few available Omics analyses of early stages of berry development and seed formation.

#### Parthenocarpy

In parthenocarpic fruits, the stimulus of pollination is sufficient to trigger fruit set (Dokoozlian, 2000). Since the ovary is able to enlarge and form a berry without ovule fertilization, there is no seed in the fruit (Varoquaux et al., 2000). Until now, few parthenocarpic grape cultivars have been described. Of these, cv. Corinto Bianco (CB), a somatic variant of the seeded cv. Pedro Ximenez (PX), constitutes a good model to study seed formation (Vargas et al., 2007). To understand the molecular differences between CB and PX genotypes, flowers at 1 and 10 days pre-anthesis were transcriptionally compared using microarray (Royo et al., 2016). The analyses allowed the identification of 1958 differentially expressed genes between CB and PX. Interestingly, several genes that are specifically expressed in reproductive organs were down-regulated in CB. Processes such as cell wall biosynthesis, cytoskeleton biogenesis, vesicular transport, signaling through G proteins or phosphatidylinositol, among others, were enriched. Also, 14 single-nucleotide polymorphisms (SNP) were identified between both genotypes, which could explain the parthenocarpy phenotype (Royo et al., 2016). Considering that microarrays deliver limited information, a suitable approach to analyze the different stages of development in more detail, would be using RNA-seq technologies, in order to gain further insights into the understanding of seed development and to generate new parthenocarpic varieties.

#### Stenospermocarpy

During stenospermocarpy, pollination and fertilization occur normally, but a few weeks later, the embryo and/or the endosperm abort and the berries that have been generated possess just traces of seed (Varoquaux et al., 2000). It has been demonstrated that stenospermocarpy occurs in several seedless varieties, and is stable and unaffected by environmental factors (Zhang et al., 2013). However, little is known about the molecular mechanisms that underlie stenospermocarpy in grapes. The most accepted hypothesis proposes the existence of a dominant regulator gene called *Seed Development Inhibitor* (*SDI*), which could control three other recessive genes (Bouquet and Danglot, 1996). Different studies based on quantitative trait locus (QTL) analysis have reported a main QTL in linkage group 18 (LG18) (Doligez et al., 2002; Cabezas et al., 2006; Mejía et al., 2007; Costantini et al., 2008), which could explain between 50 and 70% of the seedlessness phenotype in grapes; LG18 could be considered as the *SDI* locus trait. In this context, *VvAGL11* (MADs BOX transcription factor) was *in silico* mapped to the *SDI* locus and it has been proposed as the main functional candidate gene for seedlessness (Mejia et al., 2011). In fact, it was demonstrated that the silencing of a *VvAGL11* homologous gene in tomato (*Solanum lycopersicum* L. cv Micro-Tom) generates fruits with few or rudimentary seeds (Ocarez and Mejia, 2016). Based on genome sequencing data, it is known that in the stenospermocarpic variety cv. Thompson Seedless, the *VvAGL11* gene has an insertion of 15 bp in the 5′UTR, which could be the cause of the seedless phenotype (Di Genova et al., 2014). In addition, in cv. Sultanine Monococco, which is also a seeded variety of Thompson, the *VvAGL11* transcript level is higher in comparison with the seedless variety (Ocarez and Mejia, 2016), supporting the hypothesis that this gene is one of the main regulators of seed formation in grapes.

On the other hand, due to abnormal ovules may be formed during flower development before or after meiosis, through either the abnormal development of the nucellus or ovule integuments, or the degeneration of the egg in the embryo sac (Ebadi et al., 1996). In order to determine the molecular bases of this phenotype, flowers from cv. Thompson Seedless and cv. Thompson Seeded, a spontaneous mutant with seeded berries, were compared through suppression subtractive hybridization (SSH) (Hanania et al., 2007, 2009). The results demonstrated that *ch-Cpn21*, a gene that encodes for a chloroplastic chaperonin, is repressed in developing flowers of cv. Thompson Seedless. Likewise, the silencing of this gene in tobacco plants (*Nicotiana benthamiana*) and tomato induces seed abortion (Hanania et al., 2007). The use of somatic variants in combination with current transcriptomic technologies, would be very promising in the study of stenospermocarpy, helping to discover new genes playing important roles in seed abortion.

Based on the studies of Costenaro-da-Silva et al. (2010) and Nwafor et al. (2014), several genes have been associated with early stages of grape berry development. These include *VvUBP1*, a heterogeneous-nuclear ribonucleoprotein, *VvFS41*, a putative S1-like ribosomal protein involved in mRNA processing and synthesis of proteins related with cell division during the first days of berry development, *VvERF1* and *VvERF9*, which encode for transcription factors related to several developmental processes, *VvDOF1*, possibly related to seed development, and *VvRIP1* and *ABI3*, which have been related to hormone signaling, among others. Many of these genes have pleiotropic effects, so it is difficult to estimate their specific molecular contribution to the stenospermocarpy phenotype. Some of them could be involved in this process, but their functional characterization is needed to test this hypothesis.

#### Non-characterized Mechanisms of Seedlessness

A demonstrated way to produce seedlessness is the exogenous application of GAs before bloom or during anthesis. It is believed that GAs promote seedless grapes by inhibiting pollen germination, allowing unfertilized ovules to enlarge and form fruits, as occurs in parthenocarpy (Kimura et al., 1996; Cheng et al., 2013). However, another study suggests that exogenous GAs interfere with seed development, as described in stenospermocarpy (Cheng et al., 2013). So, the mechanism involved in this response is not clear. A transcriptional analysis by RNA-seq was performed in GA_3_-treated flowers of the seeded cv. Kyoho and a comparison with non-treated flowers was carried out (Cheng et al., 2015). This study demonstrated that GA_3_ application modifies the expression profile of genes related to developmental processes, such as cellular metabolism, biosynthesis of different metabolites, stress response, transport, etc. Also, changes in the expression of genes related to flowering, fruit, and embryonic development were found. Within the genes possibly related to seedlessness, the *Pelo* gene, whose mouse homolog has a role in meiosis and causes embryonic lethality (Adham et al., 2003), was repressed after GA_3_ treatment, and was correlated with seedlessness in grapes (Cheng et al., 2015). The *Pelo* gene probably has conserved roles across several species. However, deeper functional studies are needed to corroborate this information in plants and to determine if this gene does indeed fulfill a role in seed development, and more studies are needed to correlate any transcriptional changes with particular phenotypes. Recent studies have demonstrated that reactive oxygen species (ROS) are present throughout the entire seed’s life cycle (Jeevan Kumar et al., 2015). In fact, the oxidative damage induced by an imbalance in plant redox homeostasis can affect normal seed development, leading to abortion (Cheng et al., 2013). Pathways related to ROS scavenging and detoxification are significantly affected after GA_3_ treatment (Cheng et al., 2015). So, probably, exogenous GA application generates physiological changes that could induce seedless fruits through a ROS-related mechanism, but further research is needed to understand the role of ROS regarding the presence or absence of seeds. Naturally occurring seedlessness could be the result of a series of coordinated transcriptomic switches that cause a global reprogramming of the cell. To date, little is known about the seedless phenotype in grapevines, presenting a great challenge for researchers. The best model for understanding seedlessness is to compare somatic variants (seeded versus seedless) through global approaches, since they have the same genetic background and could be used to discover new genes involved in this phenotype. Even though somatic variants are rare in nature, it is clear that these comparisons are much more informative than the use of two different varieties.

### First Stage of Grape Berry Development

The first stage of grape berry development (stage I) is initiated with fruit set. During the first 2 weeks, berry size increases markedly as auxin and GAs directly promote cell division and enlargement (Ojeda et al., 1999; Bottcher et al., 2010; Fortes et al., 2015). Tartaric, malic, and other organic acids, along with different phenolic precursors such as tannins and hydroxycinnamates, are synthesized, modifying the organoleptic properties of the berries (Deluc et al., 2007). Besides, minerals, micronutrients, and aroma-related compounds are present. Transcriptomic analysis of young berries in cv. Shiraz revealed an enrichment of hormone signaling responsive transcripts, suggesting that hormone-controlled metabolic pathways are highly active in early stages of development (Sweetman et al., 2012). During this stage, GAs are the key regulators of fruit set, cell division and cell expansion (Fortes et al., 2015). RNA-seq analysis of cv. Centennial Seedless berries treated with GA_3_ (12 days after flowering), revealed a repression of an abscisic acid (ABA)-response element binding factor (ABF) and ethylene response factors (ERFs) (Chai et al., 2014). Showing the occurrence of both GA_3_–ABA and GA_3_–ethylene crosstalk. The role of jasmonic acid (JA) in grapes remains unclear, but, as has been demonstrated in potato (*Solanum tuberosum*) leaves, it might stimulate cell division (Ravnikar et al., 1992). In grape, high levels of JA are present during the first stage, which then decrease in mature berries (Fortes et al., 2015). A proteomic study in cv. Muscat Hamburg has reported abundant levels of chloroplast lipoxygenases (LOX), enzymes that provide intermediates for JA biosynthesis during green berry development, followed by a decrease when berries reach a size of 15 mm (Martinez-Esteso et al., 2011). In the case of auxins, it has been proposed that they have a role in fruit growth delaying ripening (Fortes et al., 2015). In berry flesh of cv. Kyoho, a high concentration of auxins has been reported, in particular of indole-3-acetic acid (IAA) during the beginning of stage I, with a rapid decrease at the end of this stage and throughout stage II, consistent with the high rate of cell division observed in the first stage (Zhang et al., 2003). Considering that the final number of cells in the grape berries is defined in the first stage of development (Dokoozlian, 2000), the interaction between hormones regulating cell division is key to cluster progress, and might be an interesting target in studies aimed at improving yield.

### Second Stage of Grape Berry Development

#### Main Changes in Metabolites during Stage II

The second stage of grape berry development (stage II) is a lag phase, where the rate of increase in both fresh and dry weight is very low. At the end of this stage, veraison occurs, which is the transition from the second to the third stage of berry development, and is considered the onset of ripening. Different physiological and biochemical changes take place during veraison, of which anthocyanin synthesis and sugar accumulation are the most characteristic and important processes. In fact, anthocyanins are one of the main pigments present in colored grape berry skins (Souquet et al., 1996), while sugar content is widely considered one of the most important properties that define ripening (Guelfat-Reich and Safran, 1971; Jayasena and Cameron, 2008).

Depending on the cultivar, five types of anthocyanins are frequently found in *V. vinifera*, which are associated with organoleptic properties such as color (in the case of red wine), bitterness, astringency and also as antioxidant molecules with beneficial effects on human health (Dixon et al., 2005). Anthocyanin biosynthesis occurs through the phenylpropanoid pathway, in which two types of genes are involved: structural genes, encoding for biosynthetic enzymes, and regulatory genes, which are associated with temporal and spatial regulation of the structural genes (Deluc et al., 2007). Both, structural and regulatory genes are present in colorless and colored grapevine cultivars, but in the case of white cultivars, color is not expressed due to multiallelic mutations in *MybA1* and *MybA2*, that prevent the transcription of these two important positive regulators of the phenylpropanoid pathway (Kobayashi et al., 2004). In the case of red cultivars, MYBA1 and MYBA2 transcription factors control anthocyanidin glycosylation through the regulation of flavonoid 3-*O*-glucosyltransferase (*UFGT*) expression (Ford et al., 1998). The anthocyanin biosynthesis pathway is not only regulated by MYB transcription factors, as it is also controlled by the critical transcriptional R2R3-MYB/bHLH/WD40 (MBW) complex in grapevine (Wu et al., 2014).

*Flavonoid 3′-hydroxylase* (*F3′H*) and *flavonoid 3′,5′-hydroxylase* (*F3′5′H*) genes seem to be an important regulatory points in anthocyanin biosynthesis (Castellarin and Di Gaspero, 2007; Matus et al., 2016). Their proteins belong to the cytochrome P450 protein family and compete for a common precursor for the biosynthesis of red and blue anthocyanins, respectively (Bogs et al., 2006). Metabolic and transcriptomic analyses determined that in cv. Cabernet Sauvignon and cv. Shiraz, the *F3′5′H* gene is up-regulated, whilst that of *F3′H* is down-regulated (Degu et al., 2014). However, further studies are necessary to understand the fine regulation of the phenylpropanoid pathway, focusing on the anthocyanin branch. In this case, the use of varieties with different berry skin colors would be informative. None of the aforementioned studies consider pink varieties, which have an intermediate color between red and white varieties, and could be used to complete the overview of anthocyanin biosynthesis in a fuller range of colors.

In a recent analysis using Omics approaches to analyze ripe berry skins of five cultivars, Cabernet Sauvignon, Merlot and Pinot Noir (red cultivars), and Chardonnay and Semillon (white cultivars) (Ghan et al., 2015), several transcripts and metabolites were mapped to the phenylpropanoid pathway. A higher transcript abundance for enzymes involved in anthocyanin biosynthesis, such as phenylalanine ammonia-lyase (*PAL*), chalcone synthase (*CHS*), flavanone 3-dioxygenase (*F3H*), leucoanthocyanidin dioxygenase (*LDOX*), and *UFGT* was observed only in red cultivars. Shikimate was the most abundant metabolite in cv. Cabernet Sauvignon, which acts as a precursor for aromatic amino acid biosynthesis within the shikimate pathway (Maeda and Dudareva, 2012). This intermediary is important because it allows the transfer of the carbon skeleton into anthocyanin structures, and could become a crucial point in the study of anthocyanins in different varieties.

Sugar accumulation (mainly of glucose and fructose) is another important process that begins in veraison and continues throughout ripening. Sugar sensing mechanisms may play important roles during grape berry ripening, as they do in other aspects of plant development (Smeekens et al., 2010; Wind et al., 2010). Thus, the role of sugar is covered in the context of the third stage of berry development (see Third Stage of Grape Berry Development).

#### Hormonal Control during Stage II

ABA levels are high in young berries and then fall until veraison. A microarray analysis carried out over seven sequential points of berry development in cv. Cabernet Sauvignon, revealed that the transcript abundance of 9-*cis*-epoxycarotenoid dioxygenase (*NCED1*), the enzyme that conducts the limiting step in ABA synthesis (Tattersall et al., 2007), increases during the lag phase and peaks at veraison (Deluc et al., 2007). A similar expression pattern was shown for a gene encoding for the ABA signaling transduction protein phosphatase 2C ABI1, while the gene encoding a transcription factor of the same pathway, ABI3/VP1 (Abscisic acid Insensitive 3/Viviparous 1), showed the highest transcript abundance during lag phase (Deluc et al., 2007). Several studies have highlighted the control that ABA exerts over the biosynthesis of anthocyanins; at the transcriptional level by upregulation of biosynthetic genes, and at the metabolic level by increasing anthocyanin content (Wheeler et al., 2009; Giribaldi et al., 2010; Cramer et al., 2014). In this context, A 2-DE proteomic approach in cv. Cabernet Sauvignon showed that ABA treatment before veraison increases three proteins required for flavonoid biosynthesis: chalcone isomerase, dihydroflavonol-4-reductase, and anthocyanidin reductase (Giribaldi et al., 2010).

In non-climacteric fruits, such as grape, the role of ethylene is not fully understood due to the low levels of this hormone during development and the technical difficulties associated with its quantification (Symons et al., 2012). Nevertheless, several reports indicate a possible role of this hormone in grape berry ripening, mainly supported by the consistent presence of a small peak about 2 weeks after veraison (Chervin et al., 2004). These data are consistent with the findings of Pilati et al. (2007), who showed that the expression of the *ACC synthase* gene, involved in ethylene synthesis, increases just prior to veraison and decreases afterward, together with a peak in expression of *ACC oxidase* around veraison, which encodes for the enzyme responsible for the last step of ethylene biosynthesis.

Brassinosteroids (BR), on the other hand, are steroid hormones that have been implicated in the ripening of non-climacteric fruits (Symons et al., 2006; Chai et al., 2013). The transcript abundance of the brassinosteroid receptor 1 gene (*BRI1*) peaks in the entry to lag phase and declines thereafter (Deluc et al., 2007). The expression profile of *VvBR6OX1*, which encodes for the enzyme that converts 6-deoxocastasterone to castasterone (the bioactive BR in grapes) shows a peak of induction just prior to veraison (Pilati et al., 2007). This evidence is consistent with an increase in BR levels at veraison and the high content observed during ripening in cv. Cabernet Sauvignon berries (Symons et al., 2006). Interestingly, exogenous application of BR increases anthocyanin content leading to premature grape berry coloration, similar to the effect of ABA. The connection between the molecular pathways of BR and ABA that regulate initial events of ripening stages has yet to be clarified. Based on transcriptomic analysis of cv. Merlot berries, it has been hypothesized that BR might be an early signal for ripening, modulating ethylene content (Ziliotto et al., 2012). In this model, the small peak of ethylene could upregulate genes associated with ABA biosynthesis and then initiate all ripening-associated ABA-induced metabolic changes (Ziliotto et al., 2012).

It has been well documented that auxin has a negative role during grape berry ripening. In fact, IAA levels (the active form) remain low from veraison throughout ripening, and auxin treatments during pre-veraison inhibit ripening (Davies et al., 1997; Bottcher et al., 2010, 2011; Ziliotto et al., 2012). Two auxin carriers (an AUX1-like and a PIN1-like) are expressed before veraison, while two auxin response factors (ARFs), *ARF5* and *ARF18*, and an auxin receptor of the ABP family are expressed at pre-veraison and are then repressed during ripening (Pilati et al., 2007; Fortes et al., 2011). It has been suggested that ethylene represses auxin biosynthesis and thus regulates the balance between auxin and ABA to initiate ripening (Ziliotto et al., 2012). Probably, a network coordinated by ABA, BR, ethylene, and auxin levels are regulating the ripening stage, however, the master regulators that connect all these pathways are still unknown.

### Third Stage of Grape Berry Development

#### Main Changes in Metabolites during Stage III

During the third stage of development (Stage III), berries approximately double in size and there is a marked decrease in organic acid concentration and a dramatic accumulation of glucose and fructose (∼1 M each) in the vacuole of flesh cells (Fontes et al., 2011; Dai et al., 2013). The scientific community has gained understanding about the complexity and diversity of sugar-sensing systems, including hexokinase (HXK), protein kinases, as well as, novel molecular regulators, such as trehalose-6-phosphate (T6P) (Li and Sheen, 2016). It has been shown that the HXK enzyme, responsible for the 6-phosphorylation of glucose and fructose, plays a dual-function with both catalytic and regulatory activities and therefore, links gene expression and metabolism in plants (Moore et al., 2003). HXK-dependent signaling represses photosynthetic related-genes in the presence of hexoses, forming a repressive complex that is directly associated with the promoter regions of several genes including those that encode for chlorophyll *a*/*b* binding protein (CAB) and carbonic anhydrase (CAA) (Cho et al., 2006).

In grapevine, a genome wide analysis using the completely sequenced *V. vinifera* genotype PN40024 (cv. Pinot Noir) led to the identification of six members of the HXK family (Çakir, 2014). Four genes that encode for HXKs in cv. Cabernet Sauvignon were analyzed (Gambetta et al., 2010). The authors showed that these genes are highly regulated at the transcriptional level during berry development. Specifically, *HXK-1*, *HXK-2*, and *HXK-3*, which were induced during ripening, while *HXK-4* was repressed (Gambetta et al., 2010). Interestingly, under water deficit conditions, *HXK-4* was induced during the third stage of berry development compared to the control under well irrigated conditions, indicating that there is both genetic and environmental control of the sugar sensing mechanisms during ripening (Gambetta et al., 2010).

Protein kinases are the major components of intracellular signaling and are responsible for rapid responses to changes in the environment. *VviSK1*, a protein kinases with sugar signaling function during berry development, whose transcript was shown to be accumulated after sucrose treatments in cv. Cabernet Sauvignon suspension cells (Lecourieux et al., 2010), positively affects sugar accumulation in grape cells and controls glucose transport through the regulation of four genes that encode the hexose transporters *VvHT3*, *VvHT4*, *VvHT5*, and *VvHT6*. Moreover, during berry development, *VviSK1* transcripts decrease after the green stage and increase again after veraison, when sugar is accumulated (Lecourieux et al., 2010). Another protein kinase that may participate in sugar signaling during ripening is SnRK1 (Sucrose-non-fermentative Related kinase 1). In plants, SnRK1 receives inputs from hormones, as well as, sugar phosphates, and has been linked to several developmental processes and the control of primary and secondary metabolism, including photosynthesis and anthocyanin biosynthesis (Baena-Gonzalez et al., 2007; Nunes et al., 2013; Tsai and Gazzarrini, 2014). In grapevine, *SnRK1* transcripts accumulate continuously in cv. Cabernet Sauvignon berries from the green stage until ripening (Gambetta et al., 2010). Nonetheless, to our knowledge, the abundance and activity of this protein kinase has not been measured in grapevine berries.

Phosphate sugars are other members of the sugar signaling landscape. Among them, T6P, which is generated by primary metabolism (Lunn et al., 2014) has been recently uncovered as a signal molecule with major implications in plant growth, development, and metabolism (Van Houtte et al., 2013; Wahl et al., 2013). Transcriptomic studies had uncovered that several genes that control T6P abundance are regulated during berry development. In one of the first gene expression profile analyses using AFLP in berry samples from cv. Corvina, the authors reported a T6P-phosphatase as one of the most upregulated genes in postharvest (Zamboni et al., 2008). Moreover, Deluc et al. (2007), using the first commercially available grapevine Affymetrix, identified different profiles for genes encoding T6P-synthase, which was overexpressed in the early days before veraison, and T6P-phosphatase, which was overexpressed at postharvest. Suggesting that the abundance of T6P is highly controlled during grape berry development.

In plants, T6P is linked to sugar signaling and the control of SnRK1, which is linked to developmental processes and control of metabolic pathways, including repression of anthocyanin biosynthesis (Baena-Gonzalez et al., 2007). In grapevine, several transcriptomic studies have shown that orthologs genes of *SnRK1* and of the enzymes that control T6P homeostasis are highly regulated during berry development (Deluc et al., 2007; Gambetta et al., 2010). Therefore, the SnRK1/T6P pathway may be an important component of sugar signaling during berry development, and in this context, it remains to be studied whether the activity of SnRK1 protein kinase is actually inhibited by T6P, as found in other plant tissues. The Omics studies shown so far, using mainly transcriptomic and metabolomic approaches, have been useful for the identification of several sugar-signaling components, leading to the proposal that new mechanisms or candidate genes are involved in berry ripening. Nonetheless, specifically in the field of signaling through protein kinases, it is known that transcript accumulation is not the only, or main mechanism that influences their role and activity. In this perspective, it may be necessary to perform more protein-oriented Omics studies such as proteomics or phosphoproteomics. These are powerful technologies and could help to elucidate the importance of protein kinase signaling during berry development.

#### Hormonal Control during Stage III

Microarray and RNA-seq analyses have uncovered transcriptional reprogramming during ripening (Fasoli et al., 2012). At the onset of ripening in cv. Cabernet Sauvignon, low levels of IAA are required, while the auxin conjugate to aspartate (inactive form) concentration is high (Bottcher et al., 2010). In the case of IAA conjugate formation, the up-regulation of a gene coding for GH3.1 was found at veraison (Bottcher et al., 2010), in contrast to a decrease in GH3.3 expression (Pilati et al., 2007). On the other hand, genes coding for AUX–IAA proteins, transcriptional repressors of auxin-responsive genes, are down-regulated during ripening, while genes coding for IAA19 and IAA16 are up-regulated around veraison. Likewise, a gene homologous to *Arabidopsis* amidase *AtAMI1*, that *in vitro* synthesizes IAA from indole-3-acetamide, decreases its expression during ripening (Pilati et al., 2007) indicating a complex regulation for the maintenance of low levels of active auxin during ripening.

Related to the ethylene metabolism, the role of this hormone during ripening has not been clearly established (Chervin et al., 2004). Nevertheless, it is known that the transcript abundance of genes coding for ACC synthase decrease at veraison, while several genes coding for ACC oxidase are down-regulated and only one is up-regulated during ripening (Terrier et al., 2005). The intricate regulation of the ethylene signaling pathway during ripening seems to be more consistent and clearer during the later stage of this process. Cramer et al. (2014) assessed the transcriptome of Cabernet Sauvignon berries in the late stages of ripening using whole-genome microarrays. They reported that several positive regulators of the ethylene pathway are upregulated, including three different ethylene receptors (*VviETR1*, *VviETR2*, and *VviEIN4*) and several members of the ERF family of transcription factors. Moreover, the negative regulator of ethylene signaling, *VviCTR1* is downregulated at the transcript level during late ripening in both pulp and flesh (Cramer et al., 2014). Supporting the idea of an active ethylene signaling role during berry ripening.

Regarding BRs, it has been shown that exogenous application to grape berries significantly promotes ripening, whilst endogenous BR levels dramatically increase at the onset of ripening and then decrease (Symons et al., 2006). These results coincide with the transcript accumulation of the *VvBR6OX1* gene observed by Pilati et al. (2007), responsible for the synthesis of the bioactive BR, castasterone. In addition, a gene coding for an enzyme putatively involved in castasterone catabolism (castasterone 26-hydroxylase), leading to the inactivation of this BR, is down-regulated at ripening (Fortes et al., 2011). On the other hand, a gene related to BR biosynthesis that codes for steroid 5-alpha-reductase (*DET2*), was less expressed around veraison (Fortes et al., 2011). Diminished expression of biosynthetic genes could be associated with negative feedback regulation by increasing levels of BRs.

The ABA concentration increases dramatically during berry ripening (Coombe and Hale, 1973). Several reports suggest that this hormone plays a major role controlling color development (Koyama et al., 2010) and softness (Gambetta et al., 2010). ABA levels are directly related to changes in NCED activity (Wheeler et al., 2009), and indeed, *NCED1* transcripts peak around veraison and decrease at advanced ripening (Deluc et al., 2007). A proteomic analysis in berry skins of cv. Barbera at different stages throughout ripening revealed that the most abundant proteins belong to the ABA stress responsive elements (ASR) family, representing nearly 13% of the total protein spot volume in early ripening (Negri et al., 2008). In *Arabidopsis*, the proposed model of ABA signaling involves the protein kinases SnRK2, which act as positive elements in signaling downstream of ABA. SnRK2s interact with the negative regulators PP2C protein phosphatases that inhibit the activity of SnRK2s. Recently, Liu et al. (2016) identified eight *VviSnRK2* genes in the grapevine genome and generated a detailed co-expression network of the ABA signaling components, including transcription factors from the ABF family. They found a high co-expression coefficient of both *VviSnRK2.8* and *VviSnRK2.11* with *VviABF2*, which is an important transcriptional regulator of ABA-dependent signaling during grape berry ripening (Nicolas et al., 2014). *VviABF2* expression rises from veraison until ripening, and transcriptomic analysis of *VviABF2*-overexpressing grapevine cells allowed the identification of several co-overexpressed genes regulated by ABA (Wong et al., 2013; Nicolas et al., 2014). The regulation of the ABA signaling pathway is complex; the cellular, physiological and transcriptomic responses to this hormone change dramatically in a tissue-specific manner (Rattanakon et al., 2016), and are also cultivar-dependent (Rossdeutsch et al., 2016). Moreover, as has been mentioned, the gene families that act positively, or negatively downstream of ABA are composed of several genes, and it is plausible that sub-specialization of members in these families exists. The complexity mentioned above is a significant challenge for researchers when attempting to extract mechanisms related to ABA signaling. An important issue to address in the coming years is that of understanding the differential sensitivity to ABA across cultivars, and the crosstalk of ABA with other signals that seem to be important in berry development, such as sugars and other hormones.

## The Effect of the Environment on Grape Berry Development

Grape berries are constantly exposed to several biotic and abiotic factors that, to some extent, can affect their normal development and trigger positive or negative changes. In most cases, these factors negatively impact grape cultivation at different stages of plant and berry development during pre- and post-harvest (Armijo et al., 2016a). In this review, some of the most relevant grapevine abiotic and biotic stresses are discussed.

### Abiotic Stress

Climate change has caused significant warming in most grape-growing areas, increasing some important abiotic stresses like heat, drought and UV radiation (Teixeira et al., 2013; Keller, 2015). These stresses mainly affect phenolic metabolism and, at the same time, berry composition and development (**Figure 1**).

Changes in temperatures during vegetative grape development are associated with changes in berry harvest date (Meier et al., 2007). Studies of transcripts, metabolites and proteins also show that sugar accumulation and other parameters related to color and aroma could be affected. Moderate warmer temperatures (∼25°C) lead to higher berry sugar content (Coombe, 1987), while higher temperatures (>30°C) negatively affect photosynthesis, with consequent reductions in sugar, anthocyanin, and malic acid accumulation, followed by a decrease in berry size and weight (Sadras and Moran, 2012; Teixeira et al., 2013; Rienth et al., 2016; de Rosas et al., 2017). Sugar and organic acid metabolism are desynchronized in ripening grapevine fruits at high temperatures, and secondary metabolism is diminished due to the transcriptional repression of their respective genes (Rienth et al., 2016; de Rosas et al., 2017). Thus, high temperatures are a negative regulator of berry development at ripening, but the mechanism behind this is still not clear. Integrated global analyses are required to identify the possible genes associated with the changes in the corresponding physiological traits.

In general, *V. vinifera* is considered as a salt and drought tolerant species (Tattersall et al., 2007). However, stress caused by water availability is having progressively more impact, due to can generate significant effects on grapevine cultivation. In response to salinity and drought, plants intensify the synthesis of ABA, which is transported to the aerial organs, inducing changes in the expression of genes related to their acclimatization (Tattersall et al., 2007; Cramer et al., 2011). Few studies have addressed salinity and drought stress in berries. For instance, it has been shown that water stress can increase berry flavonol content and affect the expression of genes involved in biosynthesis of stilbene precursors (Teixeira et al., 2013). All these analyses suggest a differential response to water limiting abiotic stresses that is cultivar dependent. Likewise, the optimal growth temperature for grapevines may vary between cultivars, and the activation of ABA and ethylene signaling pathways can differ according to their sensitivity or tolerance to drought. These responses have consequences in grapevine berries, since a common mechanism in response to stress in these organs, is the induction of the anthocyanins accumulation, which act as protective molecules.

#### The Effect of UV Radiation in Grape Berry Development

*Vitis vinifera* is often cultivated in Mediterranean climates with varied UV-B radiation dosages (Martinez-Luscher et al., 2013), and it is considered as well adapted to solar radiation due to a variety of physiological responses, mainly based on antioxidant enzyme activities and secondary metabolites. The UV-B spectrum (280–315 nm) can provoke potential damage in macromolecules, including DNA, induce ROS, and disrupt several cellular processes in all living organisms (Frohnmeyer and Staiger, 2003; Jenkins, 2009). In grapevine, several studies have been performed to discover the processes associated with the UV radiation response during berry development. In this context, *VviHY5* (*ELONGATED HYPOCOTYL 5*), *VviHYH* (*VviHY5 HOMOLOGUE*), and *VviUVR1* (the photomorphogenic factor *UV-B RECEPTOR 1*) genes were characterized (Loyola et al., 2016). In this work, the authors described that the expression of *VviHY5* and *VviHYH* differs during grape berry and inflorescence development upon exposure to low or high UV-B radiation, while *VviUVR1* expression was not regulated by UV-B. Studies performed by Carbonell-Bejerano et al. (2014) indicated that grapevine berries respond to UV-B through the activation of the phenylpropanoid pathway and the production of photoprotective compounds. The accumulation of polyphenolic compounds in the berry involves specific UV-responsive genes that induce the expression of phenylpropanoid pathway related genes and several MYB transcription factors that regulate this pathway (Matus et al., 2009; Berli and Bottini, 2013). Matus et al. (2009) demonstrated that different light conditions increase the accumulation of flavonoid compounds in grape berries, while Loyola et al. (2016) shown that high and low UV-B radiation induce flavonol accumulation in this organ. Carbonell-Bejerano et al. (2014) suggest that UV-B radiation triggers flavonol accumulation in grape berry skin of cv. Tempranillo and induces the expression of *VvFLS1* and *VvGT5*, two flavonol biosynthetic genes. Furthermore, several flavonol biosynthetic genes are regulated by the R2R3-MYB transcription factor VvMYBF1, which triggers flavonol and anthocyanin production in grape berries exposed to solar UV radiation (Czemmel et al., 2009, 2017; Matus et al., 2009; Matus, 2016). Genome-wide microarray studies performed in grape berry skins of cv. Pinot Noir exposed to UV-C light (100–280 nm), showed 238 up-regulated genes (more than fivefold), including several genes encoding for proteins related to stilbene synthesis (Suzuki et al., 2015). These authors also reported that UV-C light increases levels of phenolic compounds like resveratrol and its analogs. Similar results were observed in berries of cv. Tempranillo exposed to solar UV radiation (Carbonell-Bejerano et al., 2014). In general, several volatile compounds accumulate in grape berries during ripening, but the amount of these compounds depends on specific irradiance levels and the type of radiation (Joubert et al., 2016).

Summarizing, there are numerous studies demonstrating that light can affect anthocyanin accumulation in berry skins. Which can be explained by changes in the expression of structural genes related to the phenylpropanoid pathway, as well as regulatory genes such as those of the *MYB*, *bHLH*, and *WD40* families (Wu et al., 2014).

### Biotic Stress

In addition to abiotic stress, grape berry development can be influenced by biotic factors, such as pathogens, of which fungal and viral diseases are the most common and harmful, negatively affecting fruit quality.

#### Fungal Infections: *Botrytis cinerea* and Its Dual Effect on Berry Development

The most important fungal disease affecting grape berry development is gray mould, caused by *B. cinerea*. Grape berries are resistant to the infection until veraison, but are highly susceptible at the onset of ripening and harvest (Kelloniemi et al., 2015). As a necrotrophic pathogen, *B. cinerea* secretes lytic enzymes and phytotoxins in order to promote cell degradation (Armijo et al., 2016b). Most of the agronomically relevant grapevine cultivars are susceptible to this pathogen, leading to significant losses worldwide.

Different large-scale approaches have been carried out in order to understand the regulatory networks and processes involved in the grape berry–*B. cinerea* interaction, and to characterize how berry development is affected. Transcriptomic and metabolic analysis of cv. Marselan, comparing *B. cinerea* berries at veraison with ripe berries, revealed that the former activates an early burst of ROS, together with multiple defense responses, including a salicylate-dependent pathway, resveratrol synthesis and cell-wall strengthening. In contrast, ripe berries activate the JA-dependent pathway against the fungus (Kelloniemi et al., 2015). As a common response, both developmental stages displayed an upregulation of genes encoding WRKY transcription factors, pathogenesis-related proteins, glutathione *S*-transferase (involved in cellular detoxification), stilbene synthase and PAL (involved in phenylpropanoid biosynthesis), and production of anthocyanins and phytoalexins. Global metabolic changes in cv. Marselan induced by *B. cinerea* infection correlate the greater resistance of veraison berries with an accumulation of resveratrol and caffeic, ferulic, and chlorogenic acids (Kelloniemi et al., 2015). Also, significantly higher levels of proline, glutamate, arginine, and alanine were detected in *B. cinerea*-infected ripe berries of cv. Chardonnay, as well as, an accumulation of glycerol, gluconic acid, and succinate, mainly in the berry skin (Hong et al., 2012). A reprogramming of carbohydrate and lipid metabolism toward an increased synthesis of secondary metabolites with antioxidant properties, such as trans-resveratrol and gallic acid, was also observed by Agudelo-Romero et al. (2015) in cv. Trincadeira. During later stages of infection, energy metabolism (photosystem I supercomplex) and secondary metabolism (phenylpropanoid and stilbenoid biosynthesis) also seemed to be downregulated (Agudelo-Romero et al., 2015).

Contrary to the effects caused by gray mould on grape berries, some particular cases of *B. cinerea* infection can generate favorable effects on wine grapes, in an interaction known as noble rot. Botrytized wines are produced from grapes that have been affected by this fungus under specific environmental conditions, which are typically hot and dry. The infection produces berry dehydration, altering metabolic processes and the saprophytic microbiota (Magyar, 2011). The berry–fungus interaction promotes the accumulation of secondary metabolites that enhance wine grape composition in ripe berries. Transcriptomic and metabolic analyses of noble rot in cv. Semillon determined that anthocyanin biosynthesis is the most consistent hallmark of noble rot. In addition, the biosynthesis of terpenes and fatty acid aroma precursors increase during the infection (Blanco-Ulate et al., 2015). *APETALA2/ETHYLENE RESPONSIVE FACTOR* (*AP2-ERF*), and *NON APICAL MERISTEM/ARABIDOPSIS TRANSCRIPTION FACTOR/CUPSHAPED COTYLEDON* (*NAC*) transcription factors, were up-regulated during noble rot (Blanco-Ulate et al., 2015). Early products of the phenylpropanoid pathway are accumulated in noble-rotted berries, such as rosmarinic acid (a cinnamic acid derivative with antioxidant and aromatic properties). Also, a significant accumulation of several flavonoid glycosides and flavanones was detected, along with build ups of cyanidin-3-rutinoside, delphinidin-3-rutinoside, cyanidin-3-gentiobioside, and delphinidin-3-gentiobioside, anthocyanins that are normally scarce in white-skinned grape berries. Other aromatic compounds such as acetophenones, benzoic acid derivatives, methoxyphenols, and phenolic glycosides showed increased abundance in noble rot, together with gallic acid, a precursor of tannin biosynthesis (Blanco-Ulate et al., 2015).

In conclusion, reprogramming of secondary metabolites and hormonal pathways are common features in *B. cinerea*-infected grape berries. Additionally, it has been shown that during grape berry development, the fruit undergoes changes that facilitate fungal infection, such as fruit softening, organic acid and sugar level modifications, loss of the preformed defenses and decreased stilbene production, among others. On the other hand, noble rot also alters berry metabolism by inducing stress responses and accelerating ripening to enhance the colonization process. *B. cinerea* infections can affect the color and sugar concentration, improving wine grape composition. This effect is caused by an imbalance of hormone synthesis and perception, which in turn activates several ripening-associated pathways. However, the mechanism behind this acceleration is still under study.

#### Viral Diseases and Their Effect on Grape Berry Development

Viral diseases are also common in grapevine plantations. Infections caused by these pathogens are highly complex, due to the large number of viral agents described and the occurrence of multiple infections (Prosser et al., 2007; Martelli, 2014; Jooste et al., 2015; Naidu et al., 2015). Grapevines show no resistance against virus; instead, viruses and host plants establish compatible interactions, where pathogens spread throughout all plant tissues, unimpeded by the resistance responses, generating global cellular stress and developmental defects. However, in compatible interactions, hosts are not passive against the pathogen, and molecular, cellular and physiological responses can be observed (O’Donnell et al., 2003; Ehrenfeld et al., 2005). In general, grapevine viruses infect vegetative organs, but infections also have consequences for berry development, causing a reduction in berry setting, and delayed berry ripening (Martelli, 1993, 2014). Molecular changes during berry ripening in virus-infected grapevine plants have been less characterized than leaf symptomatology. For instance, characterization of the Grapevine leafroll-associated virus 3 (GLRaV-3) infection in the red cv. Cabernet Sauvignon revealed the presence of viral particles in berry tissues together with massive transcriptional changes, which were more pronounced during ripening (Aquea et al., 2011; Vega et al., 2011). Since this virus is restricted to the phloem (Martelli, 2014), GLRaV-3 infection could physically modify sugar accumulation, altering source–sink relationships.

Transcript profiling analyses performed in cv. Cabernet Sauvignon berries at veraison and ripening, using the *V. vinifera* Affymetrix GeneChip, revealed numerous changes in transcripts related either to viral infection or to berry development (Vega et al., 2011). About 400 genes showed differential expression between veraison and ripening, in uninfected tissues. However, only half of these exhibited such differences when the two stages were compared in infected berries. Thus, viral disease greatly modifies the transcript abundance profile during berry development. The number of differentially expressed genes in infected berries was higher during ripening (146 up- and 86 down-regulated genes) than at veraison (41 up- and 14 downregulated genes), suggesting that the former stage could be more dramatically affected by virus infection. Among the transcripts that change in infected berries, is a group related to sugar transport and metabolism, including *ATOCT2*, a carbohydrate transmembrane transporter; *ATSPS4F*, a putative sucrose-phosphate synthase; a short-chain dehydrogenase/reductase (*SDR*) family protein involved in sugar metabolism; *SUS2*, sucrose synthase 2; and *BFRUCT3*, a beta-fructosidase. In agreement with this, glucose and fructose levels also decreased during ripening in infected berries. Several genes from the phenylpropanoid pathway were repressed by viral infection during ripening, such as *CHS2* and *UFGT*, as well as genes that encode for transcription factors MYBPA1 and MYBA (anthocyanin biosynthesis), and FLS1, related to the flavonol biosynthetic pathway. These results were further supported by a decrease in total anthocyanin content and flavonol concentration during ripening in infected berries (Vega et al., 2011).

A characterization of berries of the Italian cv. Nebbiolo harboring a mixed infection of GLRaV-1, Grapevine virus A (GVA) and Rupestris Stem Pitting virus (RSPaV), showed significant differences in bud burst index, berry weight, titratable acidity, and resveratrol content when compared with uninfected berries (Giribaldi et al., 2011). In that study, a proteomic analysis revealed that mixed viral infection affects proteins related to cell structure metabolism in pulp, such as pectin methylesterase, *N*-acetyl-gamma-glutamyl-phosphate reductase, plastid movement impaired 1, phosphoglycerate kinase, polyphenol oxidase and alpha-tubulin, among others (Giribaldi et al., 2011). A thorough study carried out over three seasons, on the effects of grapevine leafroll disease (GLD) on cv. Merlot, showed that infection impacted greatly on yield, as well as on fruit quality (Alabi et al., 2016). For instance, the authors consistently found a lower fruit yield over the seasons evaluated, supporting previous conclusions that GLD negatively affects vine performance. In virus-infected cv. Merlot plants, developing green berries showed minor compositional changes in comparison to uninfected plants. However, after veraison, dramatic variations were observed as a consequence of viral disease, suggesting that the virus can affect ripening-related processes occurring from veraison onward, as previously shown for cv. Cabernet Sauvignon (Vega et al., 2011).

In general, transcriptomic and metabolic data support the observation that viral diseases delay grape berry ripening, altering several characteristic parameters associated with this stage, such as sugar accumulation and color, among others. However, more studies should be carried out in order to establish how viruses alter grapevine berry ripening, how cultivars and environmental factors interact to produce the complete symptomatology, and how these multiple cues modify berry ripening.

## Conclusion

Significant progress has been made toward understanding grape berry development, and how environmental factors can positively or negatively regulate this process. In this field, Omics platforms have been an important tool in the elucidation of the mechanisms underlying these interactions. Due to the lack of transgenic lines and suitable technologies for reverse genetics in grapes, Omics analyses have allowed us to make progress in unraveling the complex mechanisms that take place during berry development. Of the future challenges, the establishment of a robust model to assess biological questions is key. In grapes, the availability of mutant varieties and related cultivars with contrasting phenotypes is an advantage, but differences between cultivars could be more complex than expected. Therefore, global analysis should be carried out. In this context, as Omics provide numerous tools that generate huge data sets from an overall perspective, the integration of this information is the next challenge which needs to be addressed in order to understand the different processes underlying grape berry development. Systems biology deals with the integration of these data sets, advancing the way in which biological processes are studied from gene-by-gene studies toward a global perspective, where the different processes are depicted in regulatory networks. Those networks are useful in the prediction of gene function, while providing new insights into the regulatory mechanisms at a global level. The generation of robust networks to identify new regulators and genome-wide responses to environmental factors requires a vast number of data sets and the integration of multi-omics studies. In grape berries, most of the Omics studies are based on transcriptomic and metabolomic profiles; more-integrated networks are hindered by the lack of proteomic and phosphoproteomic studies. Another challenge in the bioinformatic field is the standardization and centralization of the stored data in order to facilitate the access to, and analysis of, Omics studies. Currently in grape, due to the multiple sources of data and gene annotation, there is a lack of consensus in the integrative tools available. For instance, annotation version 1 (V1) and version 2 (V2) differ in the number of annotated genes, with V2 having around 2000 new genes and 3000 putative long non-coding RNAs (lncRNA). The integration of the different annotations is a task that remains unresolved by the scientific community studying grape. Therefore, to improve our current knowledge, further Omics studies are undoubtedly necessary, yet this new data must be integrated with systems biology tools in order to comprehensively depict the associated regulatory networks.

## Author Contributions

The manuscript was written by AS, CE, GA, CI-B, EP, CM-R, CS, FP, AA, and PA-J. AS was involved in revising the manuscript critically for important intellectual content. Manuscript editing was conducted by AS and CE. Figure design was conducted by CM-R. All authors contributed, read and approved the final manuscript. PA-J made the final approval.

## Conflict of Interest Statement

The authors declare that the research was conducted in the absence of any commercial or financial relationships that could be construed as a potential conflict of interest.

## Abbreviations

ABA: abscisic acid
ABF: ABA-response element binding factor
ARF: auxin response factor
ASR: ABA stress responsive element
BR: Brassinosteroids
CAA: carbonic anhydrase
CAB: chlorophyll *a*/*b* binding protein
CB: Corinto Bianco
ERF: ethylene response factor
GAs: gibberellins
GLD: grapevine leafroll disease
GLRaV: Grapevine leafroll-associated virus
GVA: Grapevine virus A
HXK: hexokinase
IAA: indole-3-acetic acid
JA: jasmonic acid
LOX: chloroplast lipoxygenase
NCED: 9-*cis*-epoxycarotenoid dioxygenase
PX: Pedro Ximenez
QTL: quantitative trait locus
ROS: reactive oxygen species
RSPaV: Rupestris Stem Pitting virus
SDI: seed development inhibitor
SNP: single-nucleotide polymorphism
SnRK1: sucrose-non-fermentative related kinase 1
SSH: suppression subtractive hybridization
T6P: trehalose-6-phosphate
UV: ultraviolet
